# The Interaction of Streptococcal Enolase with Canine Plasminogen: The Role of Surfaces in Complex Formation

**DOI:** 10.1371/journal.pone.0088395

**Published:** 2014-02-10

**Authors:** Vinod Balhara, Sasmit S. Deshmukh, László Kálmán, Jack A. Kornblatt

**Affiliations:** 1 Department of Chemistry and Biochemistry, Concordia University, Montréal, Quebec, Canada; 2 Department of Physics, Concordia University, Montréal, Quebec, Canada; 3 Department of Biology and the Centre for Structural and Functional Genomics, Concordia University, Montréal, Quebec, Canada; Russian Academy of Sciences, Institute for Biological Instrumentation, Russian Federation

## Abstract

The enolase from *Streptococcus pyogenes* (Str enolase F137L/E363G) is a homo-octamer shaped like a donut. Plasminogen (Pgn) is a monomeric protein composed of seven discrete separated domains organized into a lock washer. The enolase is known to bind Pgn. In past work we searched for conditions in which the two proteins would bind to one another. The two native proteins in solution would not bind under any of the tried conditions. We found that if the structures were perturbed binding would occur. We stated that only the non-native Str enolase or Pgn would interact such that we could detect binding. We report here the results of a series of dual polarization interferometry (DPI) experiments coupled with atomic force microscopy (AFM), isothermal titration calorimetry (ITC), dynamic light scattering (DLS), and fluorescence. We show that the critical condition for forming stable complexes of the two native proteins involves Str enolase binding to a surface. Surfaces that attract Str enolase are a sufficient condition for binding Pgn. Under certain conditions, Pgn adsorbed to a surface will bind Str enolase.

## Introduction

This work owes its framework to an original idea and data put forward by Miles and Plow in 1985[1]. They showed that mammalian enolase on the mammalian cell surface would bind and subsequently activate Pgn to plasmin. The original data dealt with the enolase on the surface of platelets binding to Pgn from the same species [1]. Following the original observation, there has been an explosion of data showing that a mammalian-, yeast- or bacterial-cell with enolase on its surface will bind human Pgn as well as Pgns from other mammalian species (a partial but extensive list can be found in [2]). The field has been further complicated by the findings that enolases are not alone in providing targets for Pgn [3]; fructose bisphosphate aldolase [4] and glyceraldehyde-3-phosphate dehydrogenase also function in that capacity [5]. Many other cell surface proteins as well as gangliosides will function as receptors for Pgn [6].

In many ways, the picture, while complicated, is quite satisfying. By one means or another, the cell winds up with enolase on its surface; it binds and activates Pgn; the resulting plasmin is then available for degrading unwanted proteins. Platelets with surface enolase are associated with blood clots which can be subsequently digested by the bound plasmin. Migrating mammalian cells with adsorbed enolase on their surfaces can bind and activate Pgn before they pass through tight junctions; the latter are partially degraded which permits the passage of the cell. Bacteria expressing cell surface enolase can bind and activate host Pgn and thereby use the proteolytic activity to rapidly spread an infection.

**Figure 1:**
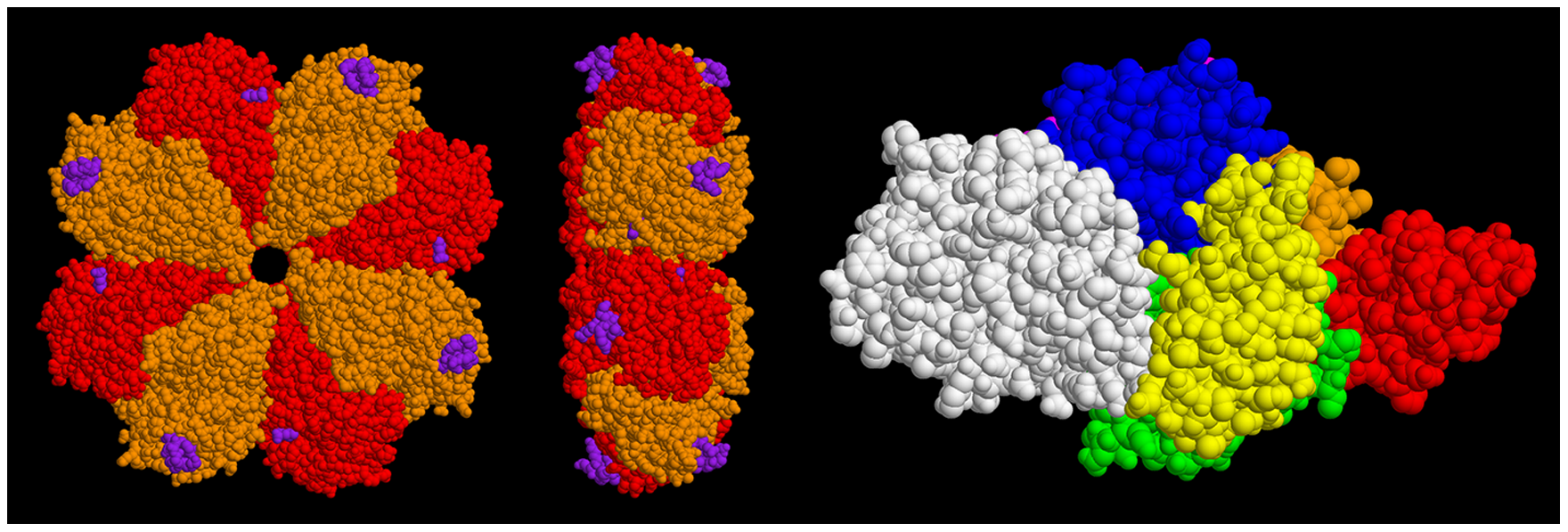
Crystal structures of Str enolase and human plasminogen. Left figures: Str enolase shown in two views. The homo-octamer is depicted in two colours. The purple atoms represent the sites where Pgn is thought to bind. Right figure: The individual domains of Pgn are colour coded. Residues 1-78 (N-Terminal Peptide), orange. Kringle 1 (79–163), red. Kringle 2 (164–249), green. Kringle 3 (250–345), yellow. Kringle 4 (346–439), blue. Kringle 5 (440–541), magenta. Preproteolytic domain (542–791), white. The figure depicts the closed form whereas it is probable that the open form (for which there is currently no structure) is that which binds to Str enolase. The two proteins are not drawn to the same scale. The dimensions of the Str enolase donut are approximately 15 nm wide and 5 nm thick. The Pgn, in contrast, is not symmetrical and consists of domains attached to, and sticking out from, a continuous string. Its largest dimensions are 10 nm×8.5 nm×5 nm. The dimensions of the two proteins will be important in determining the orientation of the two during the dual polarization interferometry experiments.

There are now crystal structures of both enolase from *Streptococcus pneumoniae* [7] (which has 93% sequence identity to the enolase from *Streptococcus pyogenes*) and human Pgn [8], [9] but not of the complex formed between the two. The two structures are shown in Figure 1.

It has been known for years that the primary binding sites in Pgn are the kringle domains which will accept C-terminal lysines as well as amino sugars and other ligands [10]–[12]. Pancholi's and Hammerschmidt's groups have shown by binding studies and X-ray crystallography that there are two sites on the streptococcal enolase that can act as co-ligands for Pgn related peptides [13] [7], [14], [15]. The first is the classical site consisting of the C-terminal dilysine. The second is an internal site spanning residues 248 to 256. Importantly, there may be other unknown sites.

We have been engaged in trying to determine the nature of the binding between the two proteins whose individual structures are shown in Figure 1. Our work has consistently shown that the interaction between the two soluble, native components is not sufficiently tight to result in an interaction that can be detected by standard biophysical techniques. We managed to show that when one of the components is denatured, the other component will bind tightly to the first[2]. We have also shown that it does not take full denaturation to demonstrate binding. Anything that shifts one of the proteins from the native to a non-native state is capable of promoting binding. *We defined non-native as a structure which does not contribute significantly to the population of molecules in the fully catalytically active, soluble state. We are now forced to revise our definition to* ***include*** *forms that are active but surface bound*.

We show in this work that either protein can bind to selected surfaces. When the first protein bound is Str enolase this is sufficient for binding canine Pgn (Pgn, 83% identity to human Pgn). In two experimental approaches surface bound Pgn would bind Str enolase. Importantly, in at least two cases, the surface bound enolase is catalytically active. We show further that the surface bound enolase is lying flat on one side of the disk thereby exposing either four or eight alternating. binding sites to Pgn.

## Materials and Methods

Ethics statement: The canine plasma used in the preparation of Plasminogen came from a commercial source: BioChemed Services, 172 Linden Dr, Winchester, Virginia, 22601 USA.

The preparation and characterization of canine Pgn has been described [2], [16]–[18] as has the preparation and characterization of enolase from *Streptococcus pyogenes* [19]. Prior to using the proteins, they were dialyzed extensively *vs.* one of several buffers after which they were centrifuged at room temperature for five minutes. Both proteins were greater than 90% pure as demonstrated by analytical ultracentrifugation, dynamic light scattering and SDS-PAGE. The buffers used were 5 mM KH_2_PO_4_, 5 mM K_2_HPO_4_, as well as 5 mM KH_2_PO_4_, 5 mM K_2_HPO_4_, 100 mM NaCl for preparation of the Pgn; the corresponding buffers for Str enolase were 50 mM Tris, 1 mM MgCl_2_, 0.1 mM EDTA, pH 7.4. For the DPI experiments, the buffer was either 10 mM phosphate or 10 mM phosphate, 100 mM NaCl. There did not appear to be any detectable influence of the salt on the interactions of the proteins with the chip or the proteins with one another. For the ITC experiments, the buffer was 10 mM HEPES, pH 7.4.

DOPG, DOPG containing 1∶100 labelled DOPE, or DOPC vesicles were prepared. Phospholipid in chloroform, in a 10 mL round bottom flask, was taken to dryness on a rotary evaporator. When dry, the phospholipids were hydrated in 10 mM HEPES, pH 7.4 or 10 mM KPi, pH 7.0 by incubating at room temperature for 1 hour. The suspensions were further dispersed with a vortex mixer. They were then extruded through 0.1 µm porous membranes (Whatman Nulclepore Track-etch) forty times. The resulting vesicles were used within 48 hours of preparation but were stable for one week. The final concentration of phospholipid was 1 mM.

All chemicals were of the highest purity available. Salts were from Fluka Chemical Co. or Sigma Chemical Co. when Fluka products were not available (Oakville, ON). The phospholipids, including the fluorescent probes, were purchased from Avanti (Alabaster, AL)

### Dual polarization interferometry (DPI)

An *Ana*Light BIO200 interferometer from FarField Group Ltd. (Manchester, UK) was used to study the interaction of Str enolase and Pgn. The technique, which has been reviewed, has not received much attention but is ideally suited to determine the nature of molecules binding to surfaces, as well as protein-protein interactions, the formation of thin films, and conformational changes occurring at the Å level [20]–[28].

Unmodified *Ana*Chips™ FB 80 from FarField Group Ltd. (Manchester, UK) with dimensions 24 mm × 6 mm were used to measure changes in the adsorbed layer. The temperature was maintained at 22°C. The flow rate through the two channels on the chip surface was controlled with a Harvard Apparatus PHD2000 pump at 10 to 20 µL/min. 200 µL of protein or phospholipid vesicles were injected into each channel. Proteins were injected at concentrations of between 1 µM and 10 µM while the phospholipid vesicles were injected at 200 µM.

Lipid bilayers of DOPG and DOPC on the chip were formed by vesicular rupture by 2 mM CaCl_2_ [29]. The bilayer was further equilibrated with flowing buffer without calcium and allowed to stabilize for at least an hour until stable values for transverse magnetic (TM) phase and transverse electric (TE) phase were obtained.

Phase changes in TM and TE were fitted to calculate the average thickness, mass, density and refractive index of the layers [29]).

### Isothermal titration calorimetry (ITC)

ITC was performed on a Microcal VP-ITC (GE Healthcare, Little Chalfont, UK). Measurements were taken at 20°C±2°. The details of its use are outlined in [2], [18], [30]. Protein solutions for both the syringe and the cell were dialyzed three times vs a minimum of 100 volumes of buffer. Both protein solutions were dialyzed in the same beaker. The dialysis schedule was 8 hours, 16 hours, 24 hours. Phospholipid vesicles were prepared in the dialysate from the second overnight dialysis. In order to see reasonable heat changes during the injections, only 15 injections were scheduled. The phospholipid in the ITC cell was 100 µM initially and received 15 large injections with buffer. This provided the buffer baseline which was subtracted from the test injections. The resulting solution in the ITC cell was mixed, the concentration calculated and then injected with 10 µM Str enolase (octamer concentration). At the end of the titration, the solution in the cell was mixed, the concentration calculated and then injected with Pgn. In evaluating the data, baselines were subtracted from the Str enolase titration into phospholipid as well as the Pgn titration into the Str enolase/DOPG solution. A one-site model was used to evaluate the titrations.

### Atomic force microscopy (AFM)

AFM measurements were performed using a Nanoscope IIIa MultiMode AFM from Veeco (Santa Barbara, CA, USA). Imaging was done in the tapping mode using V-shaped silicon nitride tips with a nominal spring constant of 0.58 N/m and a tip width of 20 nm to 60 nm. AFM measurements were carried out in environmental mode i.e. in the presence of buffer. The force applied on the samples was maintained as low as possible by continuously adjusting the amplitude set point during scanning. Image analysis was performed using NanoScope 6.14R1 software and all images are presented after flattening. Phase images give much better idea about the shape of features scanned under the liquid imaging or if the scanned material is soft and bound weakly to the substrate. Thus some of the data is presented as phase images. This clearly shows the shape of the bound Str enolase. The supported lipid bilayers of DOPG were deposited on freshly cleaved mica by vesicular rupture mechanism as explained in the DPI Materials section. The preparation of the vesicles is described [29]

### Dynamic light scattering and Fluorescence

Dynamic light scattering was performed as previously described on a Wyatt DynaPro light scattering apparatus at 20°C [2].

Förster fluorescence energy transfer was performed on a Cary Eclipse Fluorometer thermostated at 20°C [31] in the kinetic mode.

## Results

The work presented here was motivated by our inability to rationalize the large number of previous studies that showed Pgn binding to enolase on the surface of bacteria and our own study that showed that the soluble native Pgn and Str enolase would not bind to one another with a binding constant greater than 10^4^ M^−1^. We could demonstrate that binding would occur but only between non-native species. If these existed in the mammalian blood stream, they would likely be targeted for rapid degradation. This inability to rationalize was compounded by the fact that the bacterial surface-bound enolase was not covalently bound but rather interacted non-covalently with the proteins and lipids of the bacterial outermost layer. Could the difference between our non-binding results of native proteins as opposed to the binding of surface bound proteins be the difference between solution chemistry and surface chemistry [32]? If so, would an interaction between phospholipid and either of the proteins promote binding of the other? We had already shown that Str enolase would bind to azolectin (a mixture of phospholipids from soy) micelles [2]. The questions posed above are amenable to study using well developed surface techniques. Amongst these are surface plasmon resonance which we employed in our previous work [2] as well as dual polarization interferometry. We have also applied a sandwich approach (lipid vesicle/Str enolase/Pgn) to isothermal titration calorimetry which necessitated demonstrating that Str enolase was actually binding to phospholipid vesicles.

**Figure 2:**
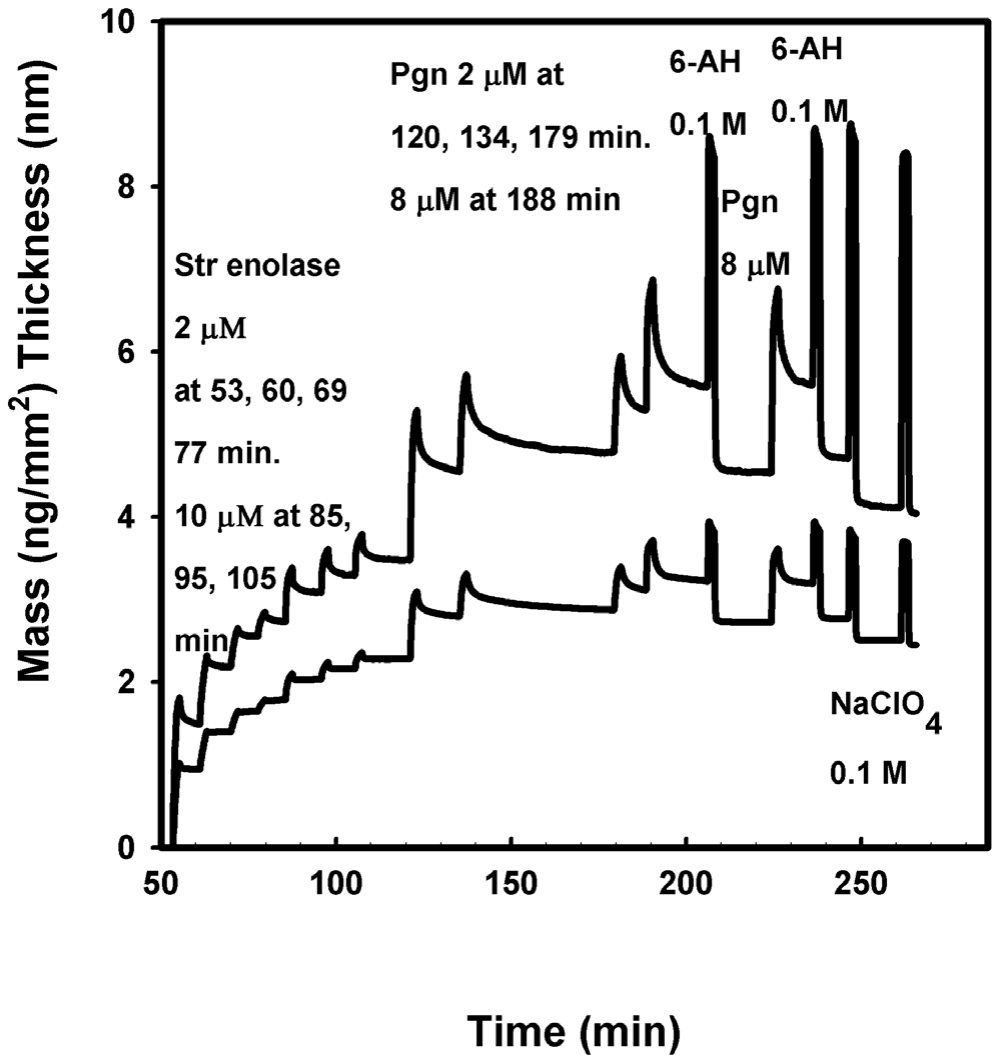
Dual polarization interferometry of Str enolase binding to silicon oxynitride followed by Pgn binding to the Str enolase. The silicon oxynitride chip was washed and calibrated in situ. The injections and their times are indicated in the figure. 6-AH ≡ 6-aminohexanoate. The lower curve indicates the calculated mass of the protein bound to the chip while the upper curve indicates the average heights of the adsorbed layers.

**Figure 3:**
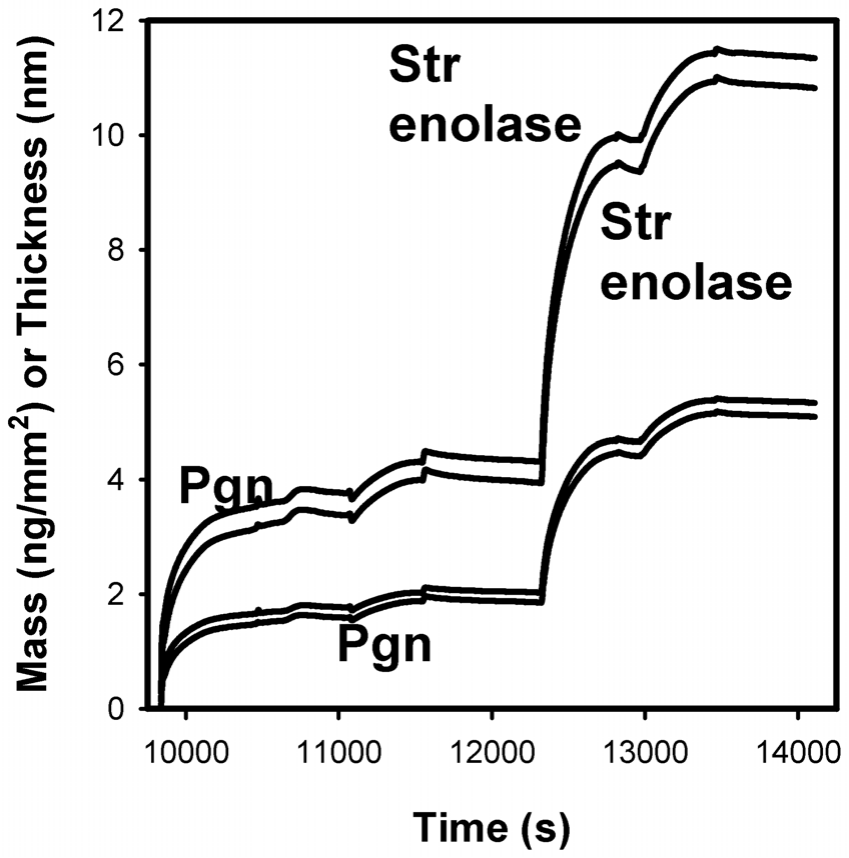
DPI of Pgn interactions with Str enolase. The silicon oxynitride chip was injected once with 200 µL 4.4 µM Pgn and the injection stopped halfway. It was then injected with another 4.4 µM Pgn which indicated that the chip was saturated. This was followed by two injections of 5.3 µM Str enolase. The two lower curves indicate the calculated average masses of the proteins bound to the chip while the two upper curves indicate the average heights of the adsorbed layers.

**Figure 4:**
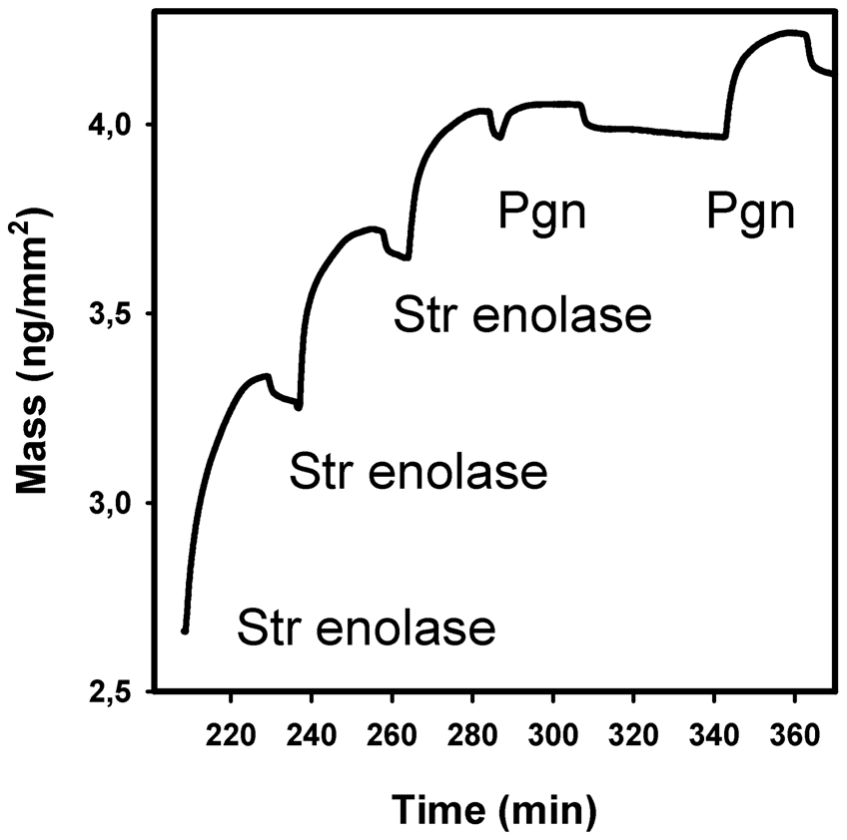
Dual polarization interferometry of Str enolase binding to DOPG bilayers followed by Pgn binding to immobilized Str enolase. The silicon oxynitride chip was saturated with DOPG. At the times shown in the figure, 200 µL of 4.1 µM Str enolase was injected into each channel. This was followed by one injection of 200 µL of 1.1 µM Pgn and one injection of 3.3 µM. The only data shown are for the mass changes of one single channel.

**Dual polarization interferometry** studies (DPI). For the purpose of understanding the data of Figures 2–4, the sensorgrams are analogous to those obtained from the related technique, surface plasmon resonance. Throughout this work we used silicon oxynitride chips to which the biomolecules bound via non-covalent interactions.

Str enolase bound to the silicon oxynitride chip and the bound protein then bound Pgn (Fig 2). In the experiment of Figure 2, we show the data for only one of the two chip channels. The increased average mass (lower curve) and the average increased height of the adsorbed mass (upper curve) were calculated values. The figure shows quite clearly that both average mass and average height of the adsorbed layer increased with the seven injections of Str enolase. Each injection took about 10 minutes after which buffer cleansed the chip, i.e. there was a buffer wash after every injection. The final injection of Str enolase indicated that the chip was saturated or almost so. Pgn was injected and it also bound.

Pgn could be partially (ca 50%) washed off the enolase saturated chip with 100 mM 6-aminohexanoate. This analogue of lysine has been known to bind to the lysine binding sites of Pgn for many years [33], [34]; and has been used to displace other tight binding biomolecules. Neither protein was washed off the chip with high salt (not shown). These two results indicate that the binding of Pgn to the Str enolase was neither exclusively through the lysine binding sites on the kringles nor mainly through electrostatic forces. Binding of Pgn to the enolase saturated chip was insensitive to sodium perchlorate, a chaotrope that has been used extensively to disrupt the solution structure of enolase [19]. This indicates that the enolase was bound to the chip via multiple interactions that helped to maintain the three dimensional structure of the protein.

Pgn bound to a silicon oxynitride chip; the second injection of Pgn indicated that the chip surface was almost saturated. The bound protein subsequently bound Str enolase (Figure 3). The sensorgrams show the data for the two channels. The adsorbed masses are shown in the two lower curves and the average heights of the adsorbed layers are shown in the upper curves. Once again, the binding of Str enolase to the Pgn saturated chip was insensitive to high salt and only partially (ca 30%) sensitive to 0.1 M 6-aminohexanoate or the chaotrope 0.1 M sodium perchlorate (data not shown).

We compare the data of Figures 2 and 3. When Str enolase was the first protein on the chip, it saturated at about 2.3 ng/mm^2^. When it was the second protein on, it saturated at 3.3 ng/mm^2^. When Pgn was the first protein on the chip, it saturated at 1.9 ng/mm^2^. When it was the second protein on, it saturated at 1.0 ng/mm^2^.The mass changes for the two proteins binding to the chip or the alternate protein were in the same range; there was a significant amount of variability between individual DPI determinations (see below). There are significant differences between the height changes found in the two figures; we could speculate on the cause for the discrepancy but have no evidence. Pgn is known to undergo a large conformational change as ligands displace lysine 50 from its internal binding site on kringle 5 [35], [36] [18], [37]; kringle 4 has also been implicated in holding Pgn in its closed position [8], [9]. If the Str enolase was binding to the lysine binding site (amongst others) it would have forced the opening of the hasp holding the Pgn in the closed conformation and thereby would have contributed to the large change in height when Str enolase bound to the Pgn bound to the chip.

The experiment of Figure 2 was repeated 10 times. We titrated the Str enolase onto the naked chip surface until such time as the chip was clearly saturated after which we titrated the Str enolase saturated chip with Pgn until it was also saturated. Since the chip had been (almost) saturated with Str enolase, it follows that the Pgn bound to the Str enolase.

Str enolase saturated at a mean value of 2.29 ng protein per mm^2^ with a standard deviation of 0.48. We translated this into the area on the chip occupied by each molecule: Str enolase/mm^2^  =  2.29 ng/mm^2^*10^−12^ mm^2^/nm^2^*10^−9^ g/ng * 6.02*10^23^molecules/mol/3.94*10^5^ g/mol **or** Str enolase per nm^2^  =  3.5*10^−3^ molecules/nm^2^. This translates into an average area per enolase of 2.9*10^2^ nm^2^/molecule.

The area of St enolase obtained from the crystal structure of Figure 1 looking down on the donut  =  176 nm^2^/molecule. If the molecules are standing on edge in random orientations, i.e., not close packed, the area per molecule would be similar.

The data indicated that the enolase would saturate the chip and that it occupied an area which was slightly larger than that determined from the crystal structure, i.e. the packing was close but not extremely close. The height of the bound Str enolase above the surface of the chip was 4.2 nm ± 1.1 nm for all 10 experiments. Leaving out two measurements judged to be outliers gave a value of 3.7 nm ± 0.1 nm. If we compare these values with the measured thickness of the donut in the crystal structure (ca 5 nm), it indicates that the disk is lying flat on the chip surface. We reiterate that the height data of Figure 3 show that when Str enolase binds to the Pgn surface, the change in height is about 7 nm rather than ∼4 nm; this may be related to the large conformational change occurring when Pgn goes from the closed form to the open form (See Kornblatt [38] and references therein for details).

The mass changes discussed in the previous paragraph indicated that there was not a sufficient amount of space remaining on the Str enolase saturated chip for any significant amount of binding of Pgn to barren portions of the chip. Nonetheless, when Pgn bound to the Str enolase saturated chip, Pgn bound and occupied approximately the same area as Str enolase occupied; Str enolase occupied 2.9*10^2^ nm^2^ while Pgn occupied 2.5*10^2^ nm^2^ (calculation not shown). This can only mean that the two proteins form (on average) a 1∶1 complex.

We have not tested to see whether Pgn bound to the chip can be activated but the Str enolase bound to the chip appears to be completely active when tested with its native substrate, 2-phosphoglyceric acid (data not shown).

In our previous publication we showed that Str enolase would bind to a phospholipid mixture from soybean; we extended that work here using purified lipids. If we first formed DOPG (dioleoyl phosphatidyl glycerol) bilayers on the silicon oxynitride chip, they would bind either protein. When Str enolase was the first protein on, it would bind Pgn (Figure 4). We determined the stoichiometry of binding in the experiment of Figure 4 (Str enolase/mm^2^)/DOPG/mm^2^). The area occupied by a single DOPG is 69.4 Å^2^ [39]–[41]. The area occupied by Str enolase/mm^2^  =  (∼2 ng/mm^2^ *6.02*10^23^ molecules/mol)/(394,000 ng/nmol*10^9^ nmol/mol)  =  3*10^9^ molecules Str enolase/mm^2^. The comparable area for DOPG on the chip is DOPG/mm^2^  =  (69.4 Å^2^/DOPG)^−1^ * 10^14^ Å^2^/mm^2^  =  1.4*10^12^ DOPG/mm^2^. **Accordingly the ratio of Str enolase molecules/mm^2^)/DOPG molecules/mm^2^) is 1**∶**470**.

If there were very close packing of enolase on the DOPG saturated chip the ratio would be 1 octamer/254 DOPG (DOPG  =  .69.4 Å^2^/molecule  =  6.94*10^−13^ mm^2^/molecule, Str enolase  =  176 nm^2^/molecule  =  1.76*10^−10^mm^2^/molecule). Clearly, the packing was close but not extremely so. Interestingly, when Pgn was the first protein bound to the bilayer, Str enolase would not bind (data not shown). This might indicate that there is a physiological relevance to the phenomena seen in this study.

We tested whether the Str enolase saturated chip would bind bovine serum albumin; it did not. We also tested whether the Pgn/Str enolase saturated chip would bind other proteins. There was weak binding of BSA and ovalbumin to the Pgn/Str enolase saturated chip but carbonic anhydrase did not bind. In our previous work [2], Str enolase was covalently bound to the chip. It strongly bound more Str enolase, BSA, yeast enolase, and Pgn but not maltose binding protein. The difference between the behaviour of Str enolase when covalently bound to a chip (SPR [2]) and that found with surface adsorption is significant. We also tested whether the Str enolase or the Pgn would bind to DOPC bilayers on the chip; neither protein would bind to the neutral phospholipid.

**ITC** experiments were carried out in order to either confirm or negate our DPI results. ITC showed that pure, solution phase, Str enolase showed no binding of pure, solution phase, Pgn in agreement with previous studies [2].

**Figure 5:**
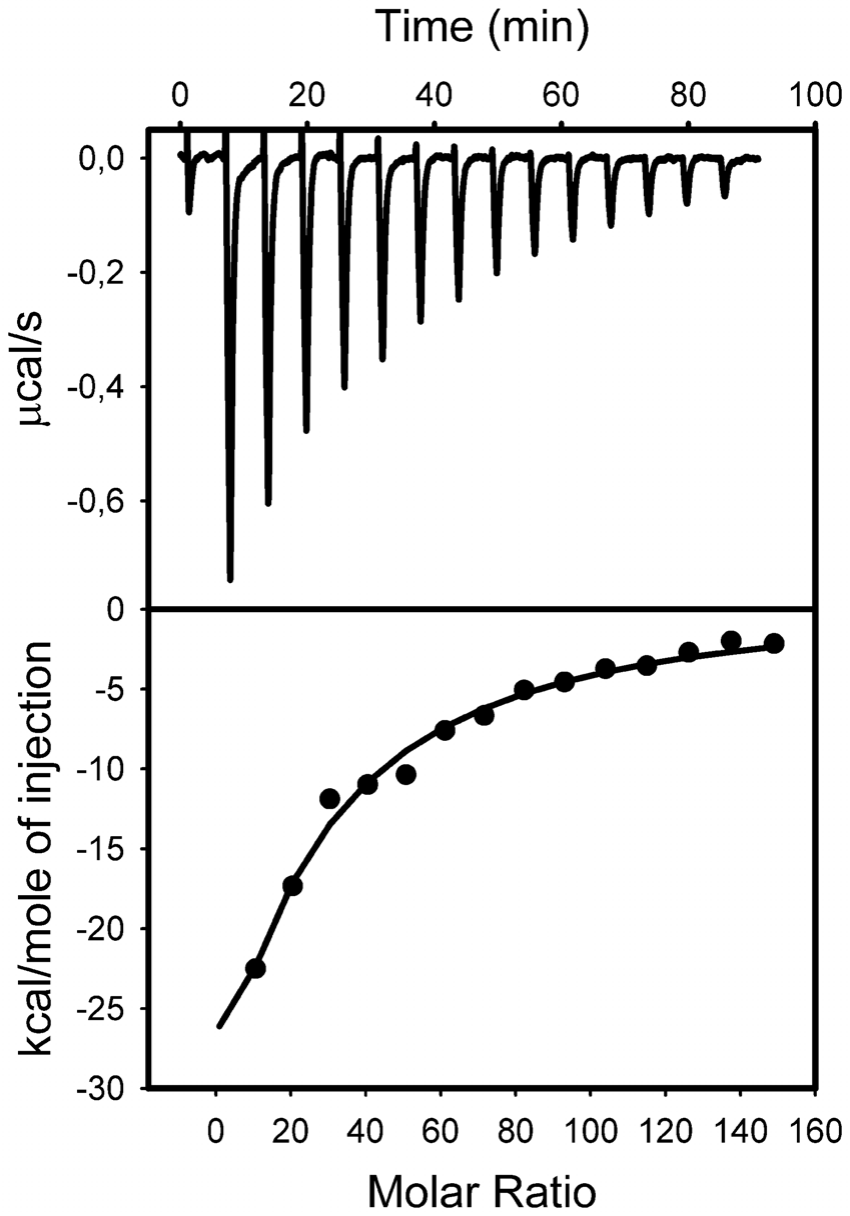
Isothermal titration calorimetry of Str enolase binding to DOPG vesicles. Titration of 0.00083 µM DOPG vesicles (86 µM DOPG) with 10 µM (octamer) Str enolase. The total final concentration of DOPG is 73.7 µM. The total final concentration of Str enolase bound is 0.24 µM.

**Figure 6:**
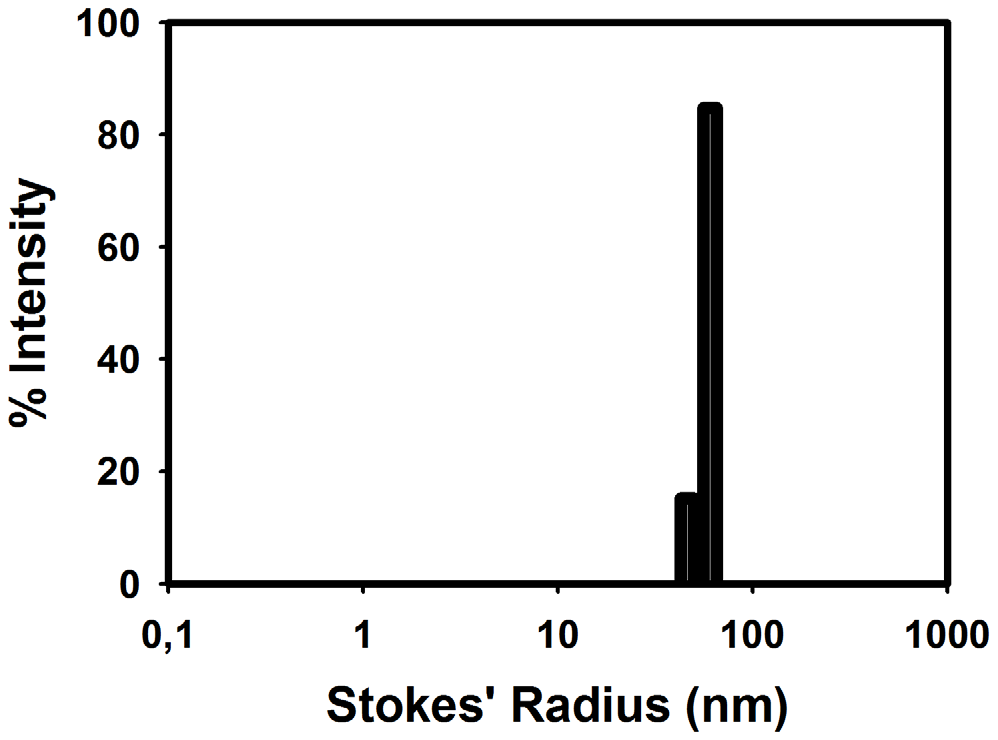
Dynamic light scattering of DOPG vesicles used for ITC of Str enolase binding. The vesicles were representative of all those used in this work. DOPG vesicles were formed from a 1 µM. The Stokes' radius of the vesicles is 58 nm.

ITC (Figure 5) and DLS (Figure 6) combined showed that Str enolase would bind to the negatively charged DOPG vesicles with a radius of 55 nm (Figure 6). How many Str enolase bound to the outer layer of a DOPG vesicle? We modelled the vesicles as spheres with outer diameter of 110 nm (1100 Å). The outer surface area of the sphere was 3,801,000 Å^2^ while the inner surface was 3, 320, 000 Å^2^ for a total area of 7.12E6 Å^2^. The surface area occupied by the DOPG headgroup is 69.4 Å^2^. There were therefore 103, 000 DOPG per vesicle. Since the DOPG concentration in the cell was 73 µM, it follows that the vesicle concentration was 7.08E-4 µM. The octamer concentration at the end of the titration is 0.11 µM. Accordingly, the ratio of the two was 155 Str enolase per vesicle. Since the outer leaflet of the vesicle contains 54, 700 DOPG molecules, **each Str enolase covered on average 353 molecules of DOPG on the outer leaflet of the vesicle**.

The value of 353 should be compared to the value of 470 obtained by DPI. The difference between the two techniques was not significant considering that the vesicles were not perfect spheres and that they undoubtedly contained invaginations which added to their surface but did not show up in the calculation of their Stokes' radius and diameter. Once again, the packing was fairly tight and was in the acceptable range in terms of the octamer lying down on the surface of the lipid. The thermodynamic parameters derived from the data of Figure 5 yielded a Ka of 1*10^5^ M, ΔH was -3*10^5^ kcal/mol and ΔS was -980 cal/mol/K. These thermodynamic values were not valid calculations since they did not represent the equilibrium between defined sites on the surface of the vesicles.

**Figure 7:**
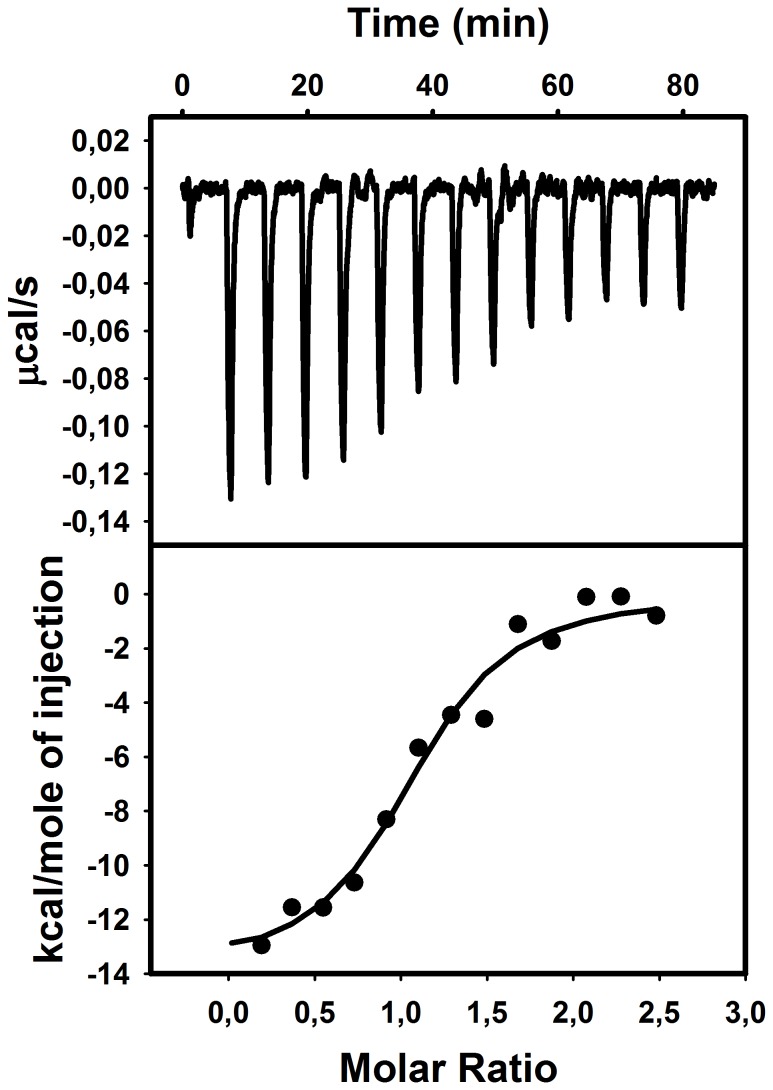
Isothermal titration calorimetry of Str enolase coated DOPG vesicles with Pgn. Titration of 73.7 µM DOPG/0.138 µM Str enolase (octamers) with 16.8 µM Pgn. The binding stoichiometry is 1.0 Pgn per Str enolase octamer.

Titration of the DOPG/Str enolase mix with Pgn resulted in formation of a 1 Pgn:1 Str enolase protein complex. (Figure 7). This agreed with the data obtained by DPI. There the stoichiometry was close to 1 Pgn:1 octamer. The ITC results were repeatable as were the DPI. The two techniques yielded the same stoichiometry. In this case the thermodynamic parameters were a valid estimation of the equilibrium between DOPG bound Str enolase and Pgn. Ka was 8.7*10^6^ M. ΔH was 1.4*10^4^ ± 0.92 kcal/mol and ΔS was -15.6 cal/mol/K.

Pgn also bound to the DOPG vesicles. Str enolase bound to this mixture with a stoichiometry of 0.75 octamers per Pgn. This does not agree with our DPI result in which Pgn bound to DOPG bilayers but the resultant complex would not bind Str enolase.

**Figure 8:**
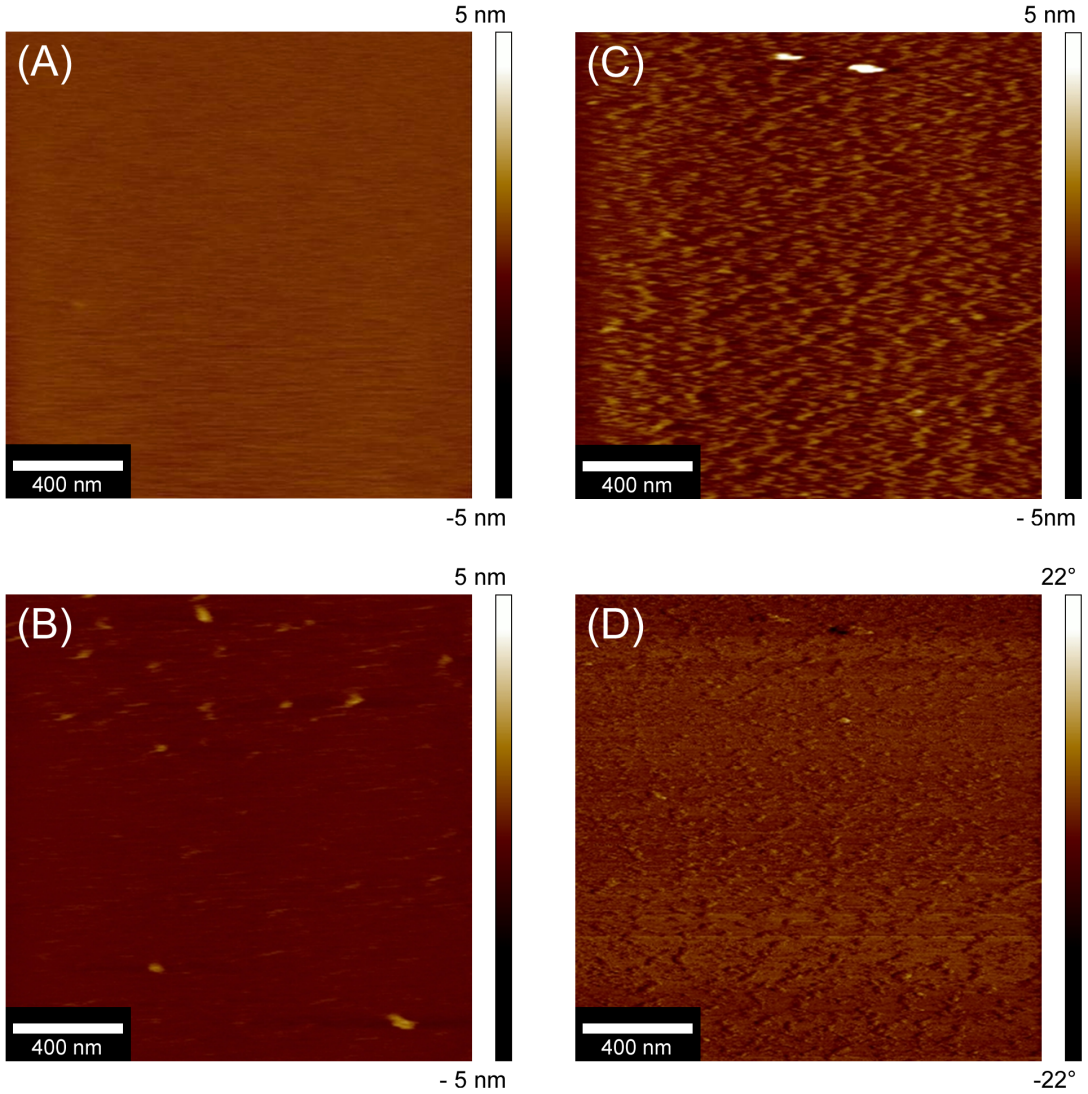
AFM of Str enolase bound to DOPG bilayers. 8A: Height image of the DOPG bilayer on the chip. The image is featureless indicating that the bilayer is well formed. 8B. Height image of Str enolase adsorbed to the DOPG bilayer. The field shows a region where the particle density is relatively low such that individual molecules can be seen. 8C. Height image of a field showing high particle density. 8D. Phase image of a field showing the uniformity of particles adsorbed to the lipid bilayer at moderate to high particle density.

**AFM** was used to study the topography of adsorbed Str enolase on the DOPG bilayers. In the best of cases, AFM can provide detailed information about the shape and size of proteins on the bilayers. It was used here to determine the orientation of Str enolase on the surface of the phospholipid bilayer. Smooth and uniform bilayers of DOPG were formed on the freshly cleaved mica. Figure 8A shows that the bilayers were both continuous and featureless. The DOPG bilayers were then incubated with 0.23 µM Str enolase for 15 minutes. AFM scanning of the bilayers incubated with Str enolase showed a range of protein density on the DOPG bilayers. In some regions the DOPG surface was completely covered with Str enolase. In other regions the density of structures was low. While there were some aggregates on the bilayer, the shape and size of the adsorbed Str enolase did not vary significantly between various regions. For the areas where the surface was less densely packed the adsorbed proteins were clearly evident in the height image of Figure 8B (light brown structures). The height and phase images of a high density region are presented in figures 8C and 8D respectively. The shape of the proteins bound to surface of the bilayers was not very clear in height images (8B, 8C). This resulted from lower deconvolution due to low lateral resolution of AFM, an effect of the finite size of the tip [42]. In the case of liquid imaging of soft samples such as proteins, the shape is much more evident in phase images (Figure 8D). It also allows for a reasonable estimation of the height of the octamer above the plane of the bilayer and a reasonable estimation of the apparent diameter of the particle but not of the details of the molecular structure.

The Str enolase appeared to be lying flat on the membrane. We measured 100 independent particles; 50 were analyzed in the phase images and 50 in the height images. The average diameter of the particles from the phase measurements was 23 nm ± 5.5 nm (Figure 8B). The average height of the particles from the height images was 1.9 nm ± 0.43 nm. (Figure 8D). The diameter of the Str enolase disk is 15 nm and the thickness is about 5 nm.[7] The particles of Figure 8B were clearly not standing on edge. The actual shape of the protein was distorted by the measurement. The AFM tip was dull and did not sharply follow the up and down contour of Str enolase. The tip had a tendency to drag the soft protein particle. Dragging coupled with not sharply following the contours resulted in exaggeration of the diameter and an underestimation of the height of the Str enolase.

This type of AFM precluded binding of Pgn to the preformed DOPG/enolase surface because it was necessary to work at low enolase density in order to obtain contrast between the smooth DOPG basement layer and the enolase; this left a significant amount of naked DOPG bilayer and we have already seen that Pgn will bind to DOPG bilayers (data not shown, DPI section above).

**Figure 9:**
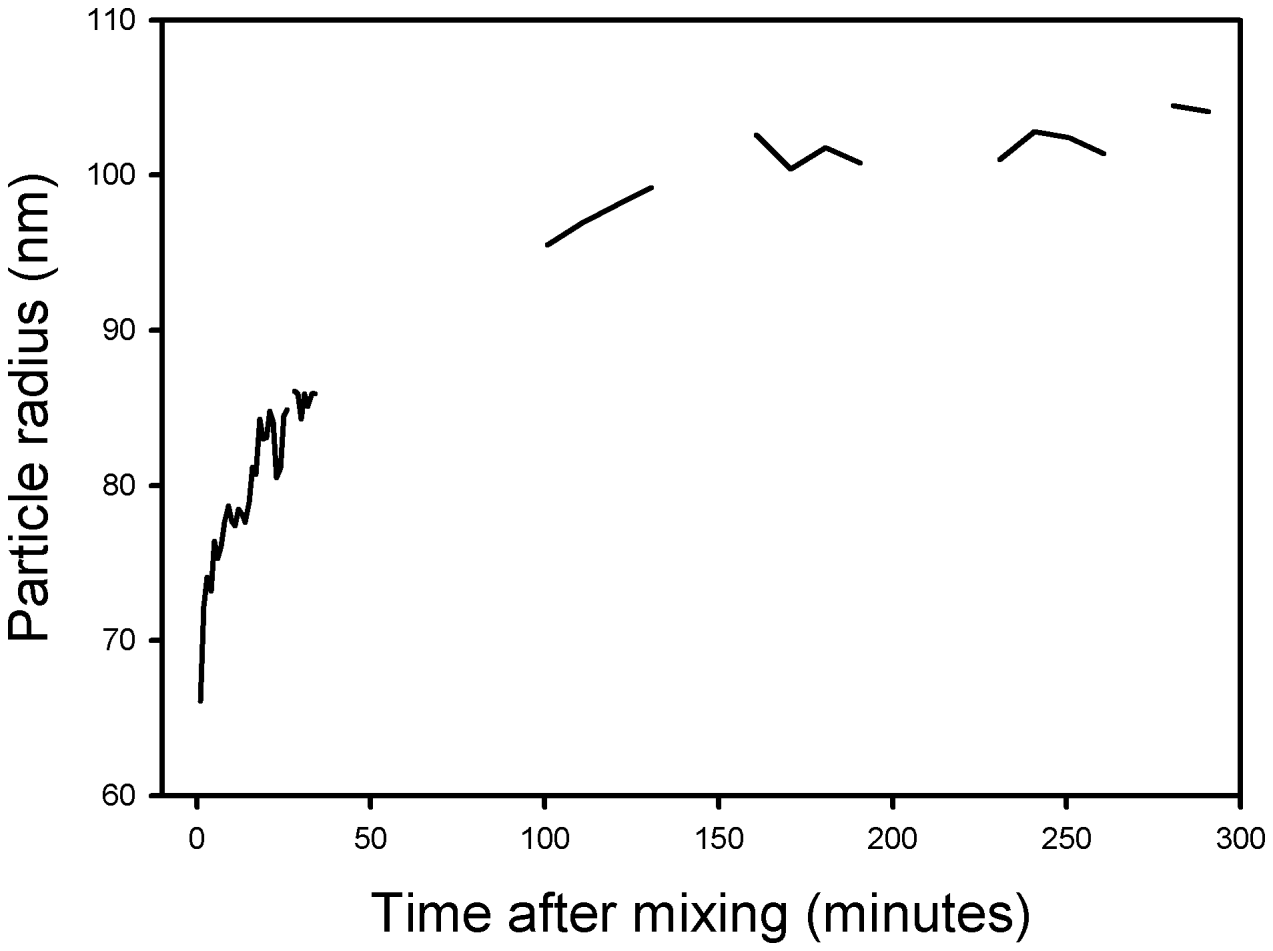
Dynamic light scattering of a mixture of DOPG and Str enolase. The DOPG was 25 µM and the Str enolase was 0.3 µM octamer, T  =  25 C. The vesicles started with a diameter of 110 nm (radius 55 nm) and on mixing with Str enolase increased over 3 hours to about 200 nm. A control suspension of vesicles did not change size.

**Dynamic light scattering and fluorescence** were used to determine what effects, if any, the Str enolase had on the DOPG vesicles. The vesicles used had the same characteristics as those used for the ITC experiments. Figure 9 shows the results of a dynamic light scattering experiment in which the DOPG vesicles were mixed with Str enolase. The structures in the DLS path clearly increased in size in a time dependent manner which indicated that the Str enolase was binding to the DOPG vesicles.

**Figure 10:**
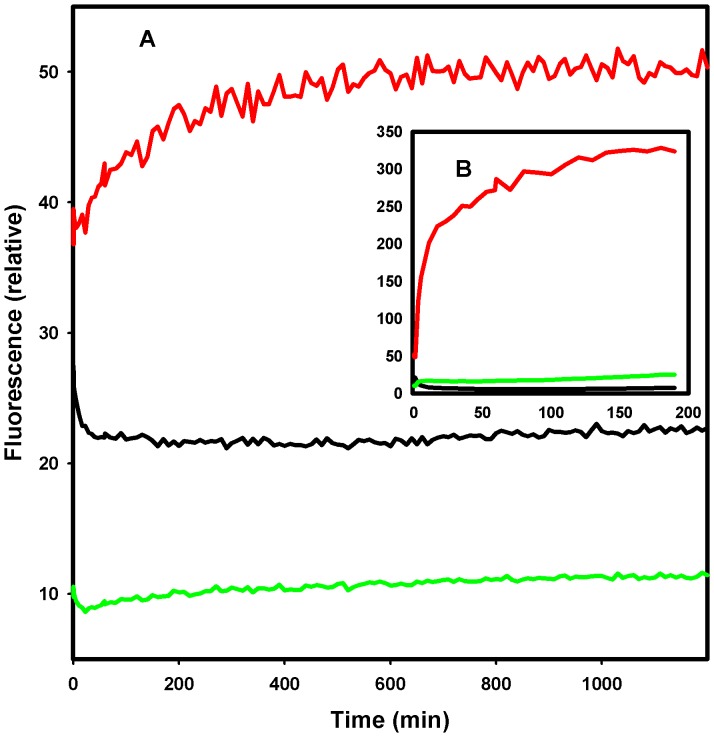
Fluorescence of (125 µM DOPG + 1.25 µM DOPE) vesicles labeled with either rhodamine or NBD in separate vesicles. The excitation wavelength was 460(NBD, maximum absorption) while the emission wavelengths were 535 nm (NBD) and 592 (rhodamine). Fusion resulting in FRET from NBD to rhodamine is indicated by a diminution of NBD fluorescence (middle curve) and an increase in rhodamine fluorescence (lowest curve). The top curve of A is the ratio of rhodamine fluorescence to NBD. At zero time the solution was brought to 1.12 µM Str enolase. In B, 1 mM CaCl2 was added to the sample. Str enolase clearly brings about a slow fusion of about 5% of the vesicles (relative to Ca).

In order to determine whether the increase in vesicle size was due to adsorption of the Str enolase to the surface of the vesicles or Str enolase promoting the fusion of vesicles, the following experiment was performed using fluorescent analogs of DOPG. NBD-labelled and Rhodamine-labelled (on the amino group of the ethanolamine) DOPE are both negatively charged like DOPG and form mixed lipid vesicles with DOPG. The vesicles we used contained both DOPG and the labelled DOPE. We used FRET from DOPE labelled with NBD to DOPE labelled with rhodamine to determine whether fusion occurred when the vesicles were mixed with Str enolase. We did two types of FRET experiments. In one, vesicles on average contained both DOPE labelled with NBD and rhodamine. In the second individual vesicles contained either DOPE labelled with NBD or DOPE labelled rhodamine. In the first instance, fusion should result in a diminution of FRET from NBD to rhodamine as the distance between the probes gets larger. In the second instance, fusion should result in an increase in FRET from NBD to rhodamine as the fused vesicles will now contain both probes. While the fluorescence data paralleled the DLS in terms of the kinetics, in both cases the data showed that some fusion occurred (Figure 10A); the small changes in Figure 10A should be compared to the large changes in Figure 10B where fusion has been promoted by the addition of Ca(II). Only the data from the vesicles containing either NBD vesicles or rhodamine vesicles labelled lipids are shown in Figure 10.

## Discussion and Conclusions

We continue to define the conditions that lead Str enolase to recognize Pgn. In our previous work we showed that anything that leads to a non-native Str enolase or Pgn would promote binding of the two [2]. Accordingly, procedures that involve Western blotting or covalent binding of Str enolase or washing with acids/bases induce non-native conformations of the two proteins and promote binding. Procedures that involve covalent labelling with a fluorescent probe may convert the protein to a non-native state and thereby promoting binding.

In the work presented here we have established that it is possible to get Str enolase to bind non-covalently to surfaces. The surface can be either in the form of a silicon oxynitride chip or a DOPG (negatively charged) bilayer but not a DOPC (neutral) bilayer. This is rather reassuring since DOPG corresponds to a major component of the Streptococcal membrane[43].

The chip-bound or bilayer-bound Str enolase will bind soluble Pgn. We have also shown that the bilayers can be either in the form of flat surfaces as are found on the silicon oxynitride chip or as vesicles. Pgn will also bind to either the silicon oxynitride chip or the DOPG on the chip. The chip-bound Pgn will bind Str enolase; the Pgn bound to the DOPG on the chip will not bind Str enolase. On either the barren chip or the bilayer, Str enolase presents the same face to the environment. If Pgn binds to the chip it potentially binds with any of several faces thereby exposing many different possible sites for binding Str enolase. If it binds to the DOPG planar surface and the binding is via one single face, that might also be the face which binds to Str enolase; this would preclude an interaction between the two proteins on the DOPG saturated chip surface. We cannot explain the discrepancy between Pgn on a vesicle surface binding Str enolase and Pgn on a flat bilayer not binding Str enolase.

Both the DPI and the AFM results demonstrated that the enolase bound to the chip or to the DOPG was lying flat on a surface of the donut. Our activity measurements showed that this enolase was completely active.

We used DOPG vesicles to reinforce the data taken with the DPI or AFM. The ITC experiments showed unequivocally that the two proteins would bind to the lipid bilayer. The stoichiometry of protein binding to lipids agreed with the DPI results. The stoichiometry of Pgn binding to protein bound lipids also agreed with the stoichiometry obtained with DPI except that Pgn on the vesicle membrane bound Str enolase. Our DLS experiments demonstrated that when Str enolase was mixed with vesicles the resulting structures increased in size, indicating binding. Fluorescence showed that the increase was accompanied by a small amount of vesicle fusion.

Does Str enolase bind to Pgn via the four lysine binding sites on the Pgn kringle domains or are other groups involved? The DPI experiments in which we treated the complexes with 0.1 M 6-aminohexanoate were designed to answer this question. The largest of the four Kds for 6-aminohexanoate on Pgn is ca 5 mM. At 0.1 M, the protein is clearly saturated but the Str enolase/Pgn complex only partially dissociates. The results with 6-aminohexanoate are similar to those of found by others [44]–[46]. It is clear that binding of Pgn to the bilayer/Str enolase mixture involves groups other than just the two carboxy-terminal lysines

In summary: We have approached the problem of our two proteins binding to one another and to phospholipid from both a qualitative and quantitative point of view. Qualitatively, we have defined conditions in which Str enolase and Pgn can bind and maintain “native”, enzymatically active, conformations. In the case of Str enolase, this requires a sticky surface. The forces that hold the Str enolase to the surface are clearly a mixture. The forces may be partially ionic but high salt does not dissociate the Str enolase from the surface. The chaotrope sodium perchlorate dissociates solution phase Str enolase [19] but has no measurable influence on surface-bound enolase. Where Pgn is bound to surface-bound enolase, the binding is not exclusively the result of the lysine binding domains on the kringles; these would be saturated by high concentrations of 6-aminohexanoate but the compound washes off only half the Pgn. The surface/Str enolase/Pgn complex is clearly the result of a diverse constellation of forces. Quantitatively, we have attempted to define the stoichiometry that is involved in the binding process as well as the reasons why the measured changes are not always those which we might have expected. Our DPI measurements involve a certain amount of variability that is a result of chip variability. All the chips were silicon oxynitride but not all chip surfaces are equally available. This gave rise to variability in the calculated masses on the chip as well as the calculated height of the adsorbed layers. The latter was additionally complicated by the fact that Pgn undergoes a very large conformational change when it binds ligands. Integrating that conformational change into the height changes seen in our DPI experiments was difficult as was relating binding to a vesicle compared to a planar bilayer. In general, the results of the several techniques that we applied in the study agreed reasonably well with one another. Where they have not agreed, we could muster hand-waving speculations but no solid reasons.
